# Epidemiology and Spatial Emergence of Anaplasmosis, New York, USA, 2010‒2018

**DOI:** 10.3201/eid2708.210133

**Published:** 2021-08

**Authors:** Alexis Russell, Melissa Prusinski, Jamie Sommer, Collin O’Connor, Jennifer White, Richard Falco, John Kokas, Vanessa Vinci, Wayne Gall, Keith Tober, Jamie Haight, JoAnne Oliver, Lisa Meehan, Lee Ann Sporn, Dustin Brisson, P. Bryon Backenson

**Affiliations:** New York State Department of Health, Albany, New York, USA (A. Russell, M. Prusinski, J. Sommer, C. O’Connor, J. White, R. Falco, J. Kokas, V. Vinci, W. Gall, K. Tober, J. Haight, J. Oliver, L. Meehan, P.B. Backenson);; Paul Smith’s College, Paul Smiths, New York, USA (L.A. Sporn);; University of Pennsylvania, Philadelphia, Pennsylvania, USA (D. Brisson)

**Keywords:** anaplasmosis, Anaplasma phagocytophilum, bacteria, tick-borne diseases, tick-borne infections, Ixodes scapularis, ticks, spatio-temporal analysis, space‒time clustering, geographical information systems, epidemiology, spatial emergence, public health surveillance, zoonoses, New York, United States

## Abstract

Human granulocytic anaplasmosis, a tickborne disease caused by the bacterium *Anaplasma phagocytophilum*, was first identified during 1994 and is now an emerging public health threat in the United States. New York state (NYS) has experienced a recent increase in the incidence of anaplasmosis. We analyzed human case surveillance and tick surveillance data collected by the NYS Department of Health for spatiotemporal patterns of disease emergence. We describe the epidemiology and growing incidence of anaplasmosis cases reported during 2010–2018. Spatial analysis showed an expanding hot spot of anaplasmosis in the Capital Region, where incidence increased >8-fold. The prevalence of *A. phagocytophilum* increased greatly within tick populations in the Capital Region over the same period, and entomologic risk factors were correlated with disease incidence at a local level. These results indicate that anaplasmosis is rapidly emerging in a geographically focused area of NYS, likely driven by localized changes in exposure risk.

Anaplasmosis is an emergent tickborne disease caused by the obligate intracellular bacterium *Anaplasma phagocytophilum* (*1*). Initially termed human granulocytic ehrlichiosis, human infection with *A. phagocytophilum* was first described in 1994 in patients from Minnesota and Wisconsin, USA (*1**,**2*). Now referred to as human granulocytic anaplasmosis or simply anaplasmosis, this infection is characterized by a nonspecific influenza-like illness marked by fever, fatigue, muscle aches, and headache (*3*). Although severe complications and death occur in rare instances, most patients recover fully after treatment with appropriate antimicrobial drugs (*4*).

Human infection with *A. phagocytophilum* has now been documented in patients in North America, Europe, and Asia, and a notable incidence has occurred in the United States (*5*). Anaplasmosis became a nationally notifiable disease in the United States during 1999, and nationwide case counts have since increased >16-fold, from 348 cases during 2000 to 5,762 cases during 2017 (*6*). Most of these infections occur in the northeastern and upper midwestern states, where well-established populations of *Ixodes scapularis* (blacklegged or deer ticks) transmit *A. phagocytophilum* in addition to the infectious agents of Lyme disease, babesiosis, and Powassan virus disease (*7**–**9*).

New York State (NYS), which is situated within the northeastern United States, to which tickborne diseases are endemic, has reported the second highest number of anaplasmosis cases of any state, closely behind Minnesota (*10**–**12*). Surveillance of anaplasmosis cases by the NYS Department of Health (NYSDOH) indicates that since the first NYS case was reported in 1994, the burden of anaplasmosis has increased substantially, accounting for a larger proportion of NYS tickborne disease cases every year (≈4% during 2010 vs. ≈11% during 2018) (*13*). Since 2015, anaplasmosis has consistently surpassed babesiosis as the second most common tickborne disease in NYS, after Lyme disease (*13*). In addition to surveillance of tickborne disease cases, the NYSDOH also conducts routine vector surveillance to monitor the dynamics of tick populations and the prevalence of tickborne pathogens, including *A. phagocytophilum*, to estimate tickborne disease risk across the state. We examined human case surveillance and tick surveillance data during 2010–2018 to assess the epidemiology, risk for pathogen exposure, and spatiotemporal emergence patterns of anaplasmosis in NYS.

## Methods

### Anaplasmosis Cases

Human anaplasmosis cases reported to the NYSDOH were analyzed retrospectively for 2010–2018 for all NYS counties, excluding the 5 boroughs of New York City (NYC). Provider-diagnosed anaplasmosis cases and positive laboratory test results for anaplasmosis were reported to the NYSDOH as mandated by NYS public health law (*14**,**15*). Both provider-reported cases and those with positive laboratory test results were investigated by NYS local health departments; clinical and demographic information for each case was entered into the NYSDOH Communicable Disease Electronic Surveillance System. Reports were assigned a case status on the basis of the 2008 Centers for Disease Control and Prevention case definition for anaplasmosis (*16*). Reports with case status of either confirmed or probable were included as cases in this study. Cases with the diagnosis of ehrlichiosis/anaplasmosis undetermined were excluded.

### Tick Collection and Testing

Host-seeking ticks were collected from publicly accessible lands across NYS during 2010–2018 by using standardized drag surveys as described (*17*). Collection sites were selected on the basis of tick habitat suitability and risk for human exposure (e.g., presence of leaf litter and hiking trails). *I. scapularis* nymphs were collected during April‒September by dragging a 1-m^2^ piece of white flannel through leaf litter and low-lying vegetation. *I. scapularis* adults were collected during September‒December by flagging a 1-m^2^ piece of white canvas over edge ecotone and understory vegetation up to 1 m high. Ticks were stored in 100% ethanol at 4°C until they were sorted by developmental stage and identified to species by using dichotomous keys, placed into sterile microcentrifuge tubes containing 100% ethanol, and stored at −20°C until DNA extraction (*18**,**19*). Individual *I. scapularis* ticks underwent total genomic DNA extraction as previously detailed and were tested for (target gene) *A. phagocytophilum* (major surface protein 2), *Babesia microti* (18S rDNA), *Borrelia burgdorferi* (16S rDNA), and *Borrelia miyamotoi* (16S rDNA) by using a quadplex real-time PCR (*17**,**20*).

### Data Analysis

We analyzed case reports meeting criteria for inclusion by using SAS version 9.2 (https://www.sas.com). Incidence rates were aggregated by NYS regions (Capital, Central, Metro, and Western), and ZIP code tabulation area (ZCTA) by using patient address and 2010 US Census population data and shapefile (*21*). We used ArcGIS version 10.7 (*22*) to map incidence at the ZCTA level. Spatial autocorrelation at the ZCTA level was determined by using Moran I analysis for each year. We determined spatial clusters by using Getis-Ord Gi* hot spot analysis (https://pro.arcgis.com) at the ZCTA level for each year. Getis-Ord Gi* analysis generated statistically significant hot spots and cold spots on the basis of the local sum of the incidence rates for each ZCTA and its neighbors within a fixed distance band at peak z-score spatial increments. We assessed temporal changes in hot spot coverage by using a 2-tailed z-test for proportions (α = 0.05).

We analyzed tick collection and pathogen testing data by using in R Studio version 1.2 (*23*) and mapped data by using ArcGIS. Tick population density was calculated for each collection site visit as the total number of target ticks (adult or nymphal *I. scapularis*) collected per 1,000 m^2^ sampled. We calculated pathogen prevalence as the proportion of ticks positive for *A. phagocytophilum* among those tested by PCR for each collection site visit and region. Temporal changes in pathogen prevalence were assessed by using a 2-tailed z-test for proportions (α = 0.05). 

We used the entomologic risk index (ERI), a measure of population density of pathogen-carrying ticks, to estimate human risk for an infected tick bite (*24*). ERI was calculated as the product of tick population density (ticks per 1,000 m^2^ sampled) and *A. phagocytophilum* prevalence at each collection site for each life stage (nymph and adult) and each year. We calculated ZCTA-level ERI as the average ERI of all sites within the ZCTA for each life stage and year. Correlation of anaplasmosis incidence and ERI at the ZCTA level was analyzed for each year by using the Spearman rank correlation. We mapped collection sites with circles sized according to ERI magnitude and then overlaid them onto the anaplasmosis incidence Getis-Ord Gi* hot spot analysis map of the corresponding year to identify common patterns in ERI and human incidence clusters.

## Results

### Anaplasmosis Epidemiology

**Figure 1:**
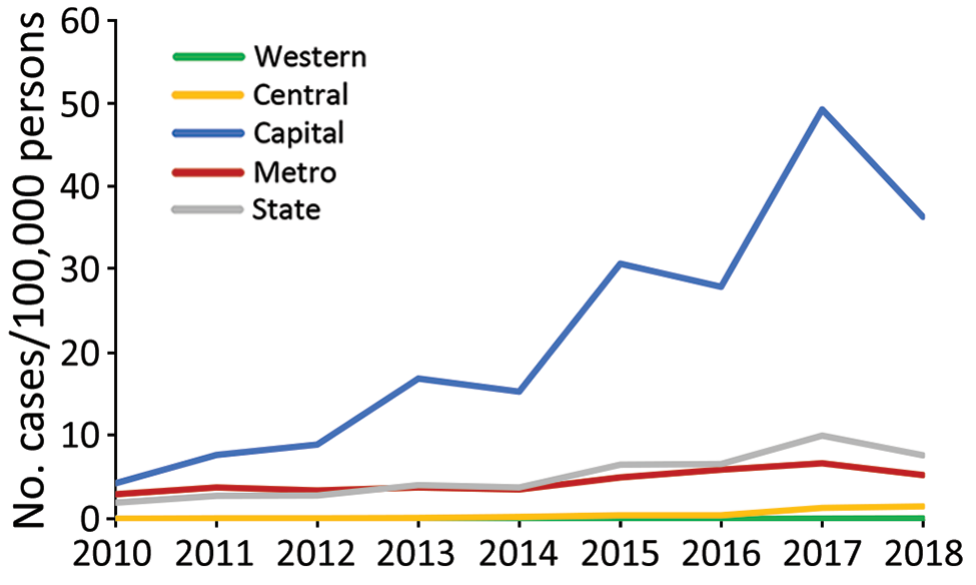
Anaplasmosis incidence by state region, New York, USA, 2010–2018.

A total of 5,146 anaplasmosis cases were reported in NYS (excluding NYC) during 2010–2018, a median of 454 cases/year (range 220–1,112 cases/year) (Table1). Statewide incidence increased 3.9-fold over the study period, from 2.0 cases/100,000 persons during 2010 to 7.6 cases/100,000 persons during 2018; peak incidence was 9.9 cases/100,000 persons during 2017. The most substantial increase occurred in the Capital Region, which showed an 8.4-fold increase, from 4.3 cases/100,000 persons during 2010 to 36.3 cases/100,000 persons during 2018; peak incidence was 49.2 cases/100,000 persons during 2017. Incidence tended to be higher in odd years, most notably within the Capital Region (Figure 1).

**Table 1:** Anaplasmosis case counts and incidence by state region, New York, USA, 2010–2018

Region	No. anaplasmosis cases, incidence/100,000 persons								
	2010	2011	2012	2013	2014	2015	2016	2017	2018
Capital	65 (4.32)	116 (7.70)	135 (8.96)	254 (16.87)	231 (15.34)	462 (30.68)	420 (27.89)	741 (49.21)	547 (36.32)
Central	1 (0.06)	2 (0.11)	2 (0.11)	3 (0.17)	5 (0.29)	8 (0.46)	8 (0.46)	24 (1.37)	27 (1.54)
Metro	154 (3.00)	196 (3.82)	178 (3.47)	195 (3.80)	183 (3.57)	257 (5.01)	304 (5.93)	345 (6.72)	273 (5.32)
Western	0	0	0	2 (0.07)	2 (0.07)	0	1 (0.04)	2 (0.07)	3 (0.11)
New York State excluding New York City	220 (1.99)	314 (2.83)	315 (2.84)	454 (4.10)	421 (3.80)	727 (6.56)	733 (6.62)	1,112 (10.04)	850 (7.67)

**Figure 2:**
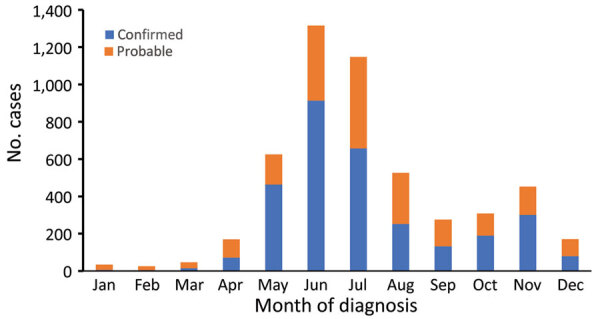
Anaplasmosis cases by month of diagnosis and case status, New York, USA, 2010–2018.

Anaplasmosis was most common among male case-patients and those identified as White and non-Hispanic (Table 2). Patients >50 years of age accounted for 72.6% of cases. Aside from fever, which is a requirement to meet confirmed or probable case status, the most commonly reported symptoms were malaise, myalgia, and chills. Rash was the least commonly reported symptom. The most common bloodwork findings were thrombocytopenia and increased levels of hepatic aminotransferases, each found in more than half of the patients. Hospitalization was reported in 35.2% of case-patients, and 0.5% (16 patients) died from anaplasmosis-related causes. Symptom onset and diagnosis occurred most often in the month of June, followed by July and May (Figure 2).

**Table 2:** Demographic and clinical characteristics of anaplasmosis case-patients, New York, USA, 2010–2018

Characteristic	% Cases
Case status	
Confirmed	60.3
Probable	39.7
Sex	
F	39.5
M	60.5
Age group, y	
0–9	2.1
10–19	3.0
20–29	4.2
30–39	7.0
40–49	11.1
50–59	20.5
60–69	24.9
70–79	18.0
>80	9.2
Race	
American Indian/Alaska Native	0.0
Asian/Pacific Islander	1.1
Black	1.0
White	95.5
Other	2.3
Ethnicity	
Hispanic	3.9
Non-Hispanic	96.1
Signs/symptoms	
Arthralgia	57.9
Chills	75.9
Headache	66.8
Malaise	90.2
Myalgia	76.8
Nausea	38.6
Rash	10.7
Rigors	19.6
Stiff neck	16.5
Laboratory findings	
Anemia	29.7
Increased levels of hepatic aminotransferases	56.6
Leukopenia	42.3
Thrombocytopenia	61.8
Outcome	
Hospitalization	35.2
Death	0.5

### Prevalence of *A. phagocytophilum*

A total of 16,743 nymphal and 27,658 adult *I. scapularis* ticks was tested for *A. phagocytophilum* during 2010–2018; a total of 721 nymphs (4.3%) and 1,789 adults (6.5%) showed positive results (Table 3). Statewide prevalence of *A. phagocytophilum* increased in nymphal and adult *I. scapularis* ticks over the study period. *A. phagocytophilum* prevalence in nymphal *I. scapularis* ticks increased in 3 of 4 geographic regions over the study period, and there was an overall statewide increase from 2.4% during 2010 to 4.5% during 2018. Statewide prevalence of *A. phagocytophilum* in adult *I. scapularis* ticks increased significantly (p<0.01) from 4.0% during 2010 to 9.2% during 2018, and we observed an increase in prevalence in all 4 regions. There was a significant (p<0.0001) 4.1-fold increase in *A. phagocytophilum* prevalence in adult *I. scapularis* ticks in the Capital Region from 2.9% during 2010 to 12.0% during 2018. Site-level ERI (*A. phagocytophilum*‒carrying ticks per 1,000 m^2^) ranged from 0 to 28.2 in nymphs and from 0 to 85.3 in adult *I. scapularis* ticks.

**Table 3:** Prevalence of *Anaplasma phagocytophilum* in nymphal and adult *Ixodes scapularis* ticks by state region, New York, USA, 2010–2018

Region	Life stage	No. (%) ticks testing positive for *A. phagocytophilum*								
		2010	2011	2012	2013	2014	2015	2016	2017	2018
Capital	Nymphs	221 (4.1)	186 (3.8)	306 (6.5)	555 (5.0)	591 (8.8)	727 (3.2)	910 (4.5)	1,223 (3.5)	1,100 (5.3)
	Adults	278 (2.9)	201 (7.0)	791 (9.9)	834 (5.9)	1,689 (5.4)	1,677 (7.0)	1,462 (8.5)	1,690 (8.6)	1,617 (12.0)
Central	Nymphs	135 (2.2)	126 (3.2)	55 (0.0)	140 (2.1)	142 (4.2)	586 (1.7)	547 (2.4)	596 (0.5)	217 (1.4)
	Adults	155 (0.0)	179 (2.2)	148 (0.7)	199 (2.0)	349 (2.0)	976 (2.8)	1,329 (1.5)	401 (5.7)	401 (5.5)
Metro	Nymphs	350 (2.0)	350 (4.9)	316 (2.2)	450 (3.3)	447 (3.8)	523 (5.0)	570 (7.7)	547 (7.7)	801 (4.9)
	Adults	300 (10.0)	350 (13.7)	350 (11.7)	518 (8.1)	544 (8.8)	625 (12.0)	1,103 (17.6)	889 (11.2)	874 (12.7)
Western	Nymphs	166 (1.2)	287 (4.9)	328 (7.3)	272 (8.1)	362 (7.2)	501 (3.0)	635 (6.1)	996 (3.4)	479 (3.5)
	Adults	276 (0.7)	395 (2.3)	691 (0.4)	646 (0.5)	681 (2.3)	1,243 (2.1)	1,444 (2.1)	1,234 (2.95)	1,122 (3.8)
New York State excluding New York City	Nymphs	872 (2.4)	949 (4.4)	1,005 (5.1)	1,417 (4.8)	1,542 (6.5)	2,337 (3.2)	2,662 (5.1)	3,362 (3.6)	2,597 (4.5)
	Adults	1,009 (4.0)	1,125 (6.7)	1,980 (6.2)	2,197 (4.5)	3,263 (5.0)	4,521 (5.4)	5,338 (6.9)	4,211 (7.2)	4,014 (9.2)

### Spatial Analysis

**Figure 3:**
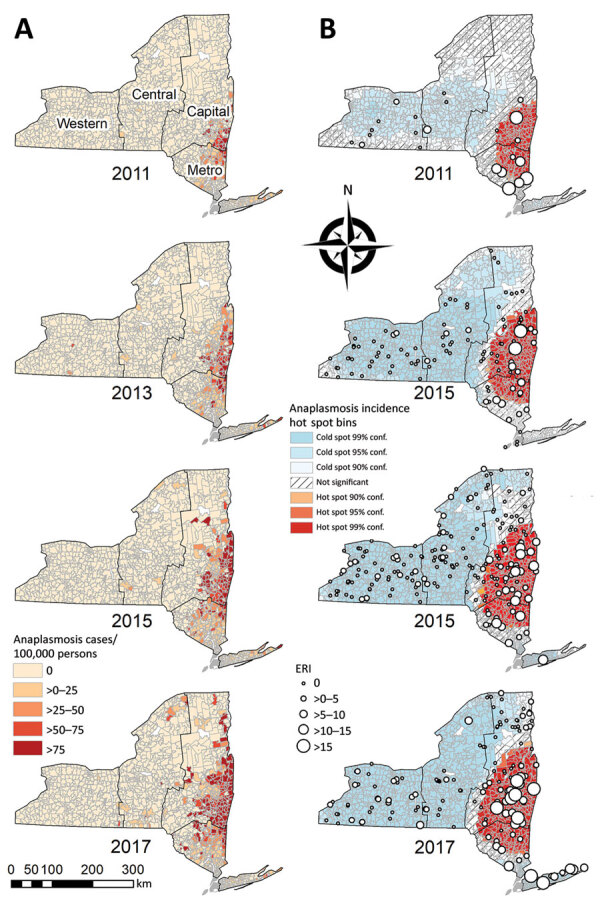
Epidemiology and spatial emergence of anaplasmosis, New York, USA, 2010‒2018. A) Incidence by ZIP code tabulation area, odd years, 2011–2017. B) Getis-Ord Gi* hot spots (https://pro.arcgis.com) and adult *Ixodes scapularis* tick ERI, odd years, 2011–2017. Conf., confidence; ERI, entomologic risk index.

Patient ZCTA was available for 5,138 (99.8%) cases. Yearly ZCTA-level incidence ranged from 0 to 1,818 cases/100,000 persons. Moran I analysis showed significant spatial autocorrelation (Moran index range 0.092–0.260; p<0.0001) of human incidence at the ZCTA level for all years, justifying hot spot analysis. Getis-Ord Gi* analysis yielded 1 statistically significant hot spot for each year during 2010–2018 (Figure 3). The 99% confidence hot spot for 2010 encompassed 14.2% of ZCTAs, 10.8% of the population, and 9.4% of the land area of NYS excluding NYC. All 3 of these factors increased significantly (p<0.0001) over the study period, and the 99% confidence hot spot during 2018 encompassed 30.0% of ZCTAs, 17.0% of the population, and 24.8% of the land area. The centroid of the hot spot moved 51.7 km north and 10.6 km west during 2010–2018. The hot spot expanded to include a larger portion of the Capital Region over the study period. Overlaying site-level ERI onto the hot spot analysis showed that collection sites that had high ERIs tended to be located within the anaplasmosis hot spot; however, multiple high-ERI sites across the Metro Region and sporadically throughout the Central and Western regions were located outside the hot spot. Nymphal ERI was significantly correlated (*r* range 0.340–0.536; p<0.05) to anaplasmosis incidence at the ZCTA level for 7 of the 9 years during 2010–2018. Adult ERI was significantly correlated (*r* range 0.291–0.695; p<0.01) to anaplasmosis incidence at the ZCTA level for all years during 2010–2018.

## Discussion

This 9-year study characterizes the emergence of anaplasmosis in NYS through the analysis of trends in human case and vector surveillance data over time and geography. Anaplasmosis poses an increasingly substantial public health risk in NYS, and the 2010–2018 time frame captures a dramatic increase in the burden of this newly emergent disease. A closer look at the changing epidemiology and exposure risk for this disease helps to elucidate when, where, and why anaplasmosis is rapidly expanding in NYS.

The basic epidemiologic characteristics of anaplasmosis cases in NYS are consistent with previous reports at the national level and are comparable with those of other tickborne diseases transmitted by *I. scapularis* ticks in NYS (*7*,*10*,*25*,*26*). Anaplasmosis, similar to Lyme disease and babesiosis, disproportionately affects White men, possibly because of residential and behavioral factors that increase the risk for tick bites in this group (*27*,*28*). The age distribution of patients shows a unimodal peak in the range of 60–69 years, similar to babesiosis but unlike Lyme disease, which shows bimodal peaks in the 5–9 and 50–54 year ranges (*27*,*28*). This finding might be related to the increased susceptibility for severe anaplasmosis infections with age, and the greater likelihood of subclinical infections in young patients (*7*,*29*). Anaplasmosis infection causes a constellation of nonspecific symptoms that mimic those of other tickborne diseases, but without a characteristic rash, such as erythema migrans, seen in Lyme disease (*27*,*28*). The hospitalization rate for anaplasmosis is higher than that for Lyme disease but lower than that of babesiosis, and the case-fatality rate of 0.5% is much lower than the 6.5% found in babesiosis patients in NYS (*26*,*27**,**29*).

The summertime peak in anaplasmosis incidence implicates *I. scapularis* nymphs, which are most active during summer months, as the developmental stage responsible for most cases, even though nymphs are approximately half as likely as adult *I. scapularis* ticks to carry *A. phagocytophilum*. This finding is consistent with other tickborne diseases and might be attributed to NYS residents spending more time outdoors during summer months and the increased difficulty of finding and removing the much smaller nymphs during the 12‒24-hour window before *A. phagocytophilum* transmission occurs (*9**,**25**,**27**,**30*).

Spatial assessment of the emergence of anaplasmosis indicates that the increase in cases is not occurring diffusely across NYS but is instead originating primarily within the Capital Region. The dramatic 8.4-fold increase in incidence in the Capital Region during the 9-year study period indicates a rapidly intensifying focal area of disease emergence. Hot spot analysis pinpoints an expanding focal area centered around Columbia and Rensselaer Counties, bordering the Hudson River to the west and Vermont and Massachusetts to the east. This area might be located within a local epicenter of anaplasmosis emergence in the northeastern states because case data for neighboring states indicate increasing anaplasmosis incidence in NYS-adjacent counties over the time frame of our study (*31*,*32*). The geographic expansion of anaplasmosis generally mimics that of Lyme disease in NYS decades earlier, with initial emergence northward along the Hudson River (*33**,**34*). However, the spread of Lyme disease more closely followed the apparent range expansion of *I. scapularis* ticks from coastal areas northward and westward across NYS, whereas anaplasmosis is less common in coastal NYS and shows a more radial expansion further inland (*35*). A similar inland radial expansion pattern was seen in the emergence of anaplasmosis in Minnesota during 1996–2011 (*36*).

The hot spot defined by this study coincides with a map of seroprevalence of *Anaplasma* species in a large sample of domestic dogs across the contiguous United States during 2011–2015 (*37*). Dogs, which are affected by the same pathogenic variant of *A. phagocytophilum* as humans, might be an excellent sentinel species in forecasting the spread of anaplasmosis, as they have been for other tickborne diseases (*38*). Many potential driving forces, including changes in land use, host density, and climate, have been implicated in the geographic spread of *I. scapularis* ticks and associated pathogens, and it is probable that a multitude of factors are shaping the spread of anaplasmosis in NYS. The rapid, geographically focused pattern of anaplasmosis emergence might also indicate recent changes in risk factors that are unique for this disease.

This study describes the changing prevalence of *A. phagocytophilum* in a large sample of host-seeking *I. scapularis* ticks collected across NYS. The overall statewide increase in pathogen prevalence over the study period, and in particular the large increase within adult *I. scapularis* ticks in the Capital Region, parallels the focal increase in human anaplasmosis incidence. The correlation of anaplasmosis ERI, which accounts for pathogen prevalence and tick population density, to yearly human incidence at the ZCTA-level, further supports the hypothesis that localized changes in exposure risk are driving emergence of this disease. However, the trends found in our tick surveillance data cannot fully explain the dramatic increase in anaplasmosis cases. Relatively high prevalence rates of *A. phagocytophilum* have been documented in *I. scapularis* ticks from the Metro Region of NYS as early as 1996 (*39*). A previous NYSDOH study found *A. phagocytophilum* in 6.5% of nymphs and 12.3% of adult *I. scapularis* ticks collected during 2003–2006 in the Hudson Valley, a region that overlaps most of the Metro Region and the southernmost part of the Capital Region as defined by this study, with no noted increase in pathogen prevalence over the study period (*17*). Clearly, *A. phagocytophilum* has been present at appreciable levels in the Metro Region tick population well before the recent increase in anaplasmosis cases, and although other tickborne diseases are endemic to this region, the Metro Region has not experienced a major emergence of anaplasmosis as seen in the Capital Region. The presence of multiple high-ERI tick surveillance sites, especially within the Metro Region, which were located well outside the anaplasmosis hot spot each year, underlines this discrepancy.

Some notable limitations of our host-seeking tick sampling might partially contribute to this incongruity, including greater vector surveillance coverage in some regions than others, increasing level of sampling effort over the study period, and repeated sampling of some locations but not others. Another explanation might be the distribution of pathogenic versus nonpathogenic genetic variants of *A. phagocytophilum*. The PCR used in this study does not distinguish between Ap-v1, a nonpathogenic variant that has a major reservoir in white-tailed deer (*Odocoileus virginianus*), and Ap-ha, a human pathogen that has white-footed mice (*Peromyscus leucopus*) and Eastern chipmunks (*Tamias striatus*) as major competent reservoirs (*40*,*41*). Studies of *I. scapularis* ticks in Ontario, Canada, which borders NYS, indicate an increase in the proportion of the pathogenic Ap-ha variant relative to the Ap-v1 variant in ticks collected after versus before 2010 (*42**–**44*). A similar shift in variant prevalence might be occurring in NYS and could be a driving force for human disease emergence. A follow-up study using genotyping to differentiate between variants of *A. phagocytophilum* in ticks collected across NYS, coupled with spatial analysis to examine changes in variant distribution over time and geography, is in progress and will hopefully further elucidate factors contributing to the emergence pattern of anaplasmosis in NYS.

The true burden of anaplasmosis in NYS is probably greater than that captured by our analysis of mandated case reporting. Anaplasmosis cases that are subclinical, self-limiting, misdiagnosed, or co-infections with other tickborne pathogens might be unreported or do not meet the strict case definition. In addition, the level of awareness of tickborne diseases among healthcare providers and the general public probably varies across NYS because tickborne diseases are hyperendemic in some regions and newly emergent in others. Resulting differences in patient behavior, provider diagnosis, and local health department reporting make estimating the true incidence of anaplasmosis a challenge, similar to what has been documented for Lyme disease in NYS (*45*). Lack of awareness can increase the likelihood of undiagnosed and untreated cases, which is especially relevant for a new and rapidly expanding disease such as anaplasmosis. Assessing disease epidemiology and clusters over time and geography enables us to pinpoint the populations at highest risk and anticipate when and where the disease will spread in the future so that public health efforts can be targeted toward populations who might benefit the most.
